# MMP9 and TYROBP affect the survival of circulating tumor cells in clear cell renal cell carcinoma by adapting to tumor immune microenvironment

**DOI:** 10.1038/s41598-023-34317-2

**Published:** 2023-04-28

**Authors:** Lingyu Guo, Tian An, Haibin Zhou, Ziyan Wan, Zhixin Huang, Tie Chong

**Affiliations:** 1 School of Medicine, Xi’an Jiaotong University, Xi’an, China; 2 Department of Urology, The Second Affiliated Hospital of Xi’an Jiaotong University, 157 West Fifth Road, Xi’an, 710000 China; 3 Department of Dermatology and Plastic Surgery, The Second Affiliated Hospital of Shaanxi University of Traditional Chinese Medicine, Xianyang, China

**Keywords:** Cancer, Computational biology and bioinformatics

## Abstract

Circulating tumor cells (CTCs) play a key role in tumor metastasis. CTCs have altered gene expression and can survive in the bloodstream. Finding the key genes whose expression are altered in CTCs could help explain the mechanism of tumor metastasis. We searched for genes differentially expressed in CTCs by analyzing four CTCs and primary tumor gene expression datasets in the GEO database. Key genes of clear cell renal cell carcinoma (ccRCC) CTCs were identified. The correlation between key genes and the immune microenvironment of ccRCC was explored. Finally, the CTCs cell model of ccRCC was constructed by in vivo screening method, and the expression of key genes was detected at the cell and tissue levels. A total of 771 DEGs were obtained. Gene enrichment analysis showed that DEGs of CTCs were mainly involved in the regulation of the tumor immune process and tumor cell apoptosis. Finally, we found 2 key genes, MMP9 and TYROBP in ccRCC CTCs. The high expression of these 2 genes predicted a poor prognosis of ccRCC, and the expression levels of these 2 genes were significantly increased in CTCs and ccRCC tissues. Our study suggested that genetic alterations in CTCs contribute to the ability of CTCs to survive in the blood by adapting to the tumor microenvironment. MMP9 and TYROBP are potential therapeutic and prognostic targets for ccRCC.

## Introduction

Renal cell carcinoma (RCC) is a malignant tumor with a high incidence in the human body, which seriously threatens people’s life and health^1^. Clear cell renal cell carcinoma (ccRCC) is the main histological subtype of RCC^2^. About one-third of patients are found to have metastasis at the time of presentation, recurrence and metastasis are the main causes of poor prognosis^3^. The development of early diagnosis and treatment of tumors has greatly reduced the mortality of malignant tumors, but there is still a lack of effective treatment for recurrent and metastatic ccRCC.

Circulating tumor cells (CTCs) are separated from the primary tumor site or metastatic tumor site^4^. CTCs enter the blood circulation through the vascular endothelium and survive in the blood, which was first observed by Ashworth in the blood of tumor patients^5^. Hematogenous metastasis is a major metastatic mode of ccRCC, and CTCs, as the seeds of tumor metastasis, play a key role in the process of ccRCC metastasis^6^. Only a small part of tumor cells that enter the blood circulation survive and metastasize in the blood circulation^7^. As CTCs are scarce in the blood, it is difficult to capture and analyze CTCs. In recent years, the development of various detection technologies has made it possible to analyze CTCs in vitro, and liquid biopsy technology has been developed to perform tumor screening, treatment response monitoring, diagnosis, and prognosis assessment through biological analysis of CTCs^8^. A large number of studies have focused on the survival and metastasis of CTCs in the blood, providing new ideas for drug treatment of tumor recurrence and metastasis.

In this study, we used the GEO database to analyze the gene expression differences between CTCs and primary tumor cells and to analyze the functions of these differentially expressed genes (DEGs) and the mechanisms that regulate the development of ccRCC. Finally, the CTCs model of ccRCC was constructed by in vivo screening method, and the expression of key genes was detected at the cellular and tissue levels, which provides potential targets for the treatment and prognosis of ccRCC.

## Methods

### Data collection and processing

We obtained four databases GSE18670, GSE50991, GSE82198 and GSE82198 from GEO database^9,10^. We list the details of the data set in Table 1. The GEO2R tool was used to process the data to obtain differentially expressed genes (DEGs) between CTCs and primary tumors. |logFC|> 1 and adjust *p* < 0.05 were used as the threshold to select DEGs for subsequent analysis. After obtaining DEGs in all four data sets, the Venn diagram method was used to include DEGs in more than three datasets for further analysis. The DAVID tool was used to perform GO and KEGG enrichment analysis on the 771 DEGs included^11^.

**Table 1:** Characteristics of the microarray databases derived from the GEO database.

Dataset ID	Cancer types	Platforms	CTC samples	Control samples
GSE18670	Pancreatic cancer	GPL570	6	6
GSE50991	Lung cancer	GPL13693	9	9
GSE82198	Colon cancer	GPL570	3	3
GSE106363	Prostate cancer	GPL15207	3	3

### Screening and analysis of key genes

The String database was used to analyze the interactions between DEGs. Cytoscape tool was used to screen and analyze key genes. We first analyzed the entire PPI network using the Cytohubba plug-in and obtained the top 10 hub genes using the MNC algorithm. Then the MCODE plug-in was used to analyze the subnetwork, and the node genes in the subnetwork with the highest score were obtained. The intersection of 10 hub genes and node genes was obtained for subsequent analysis.

### Expression and survival analysis of key genes in ccRCC

We acquired the TCGA-KIRC dataset to analyze the expression differences of key genes between ccRCC tumor tissues and normal tissues. The Kaplan–Meier Plotter tool was used to analyze the association between key genes and OS in ccRCC^12^.

### Functional analysis and immune infiltration analysis of prognostic genes

The GEPIA database was used to obtain a list of genes co-expressed with prognostic genes in ccRCC^13^. We selected the top 50 genes that correlate with each prognostic gene for GO gene enrichment analysis. The Timer database was used to analyze the relationship between prognostic genes and the level of tumor-associated immune cell infiltration in ccRCC^14^. Finally, we used the TISIDB database to analyze the association of these 2 prognostic genes with immune markers and disease immune subtypes in ccRCC^15^.

### Cells and reagents

Renal cell carcinoma cell lines 786O, OSRC2, 769P, ACHN, CAKI2, and normal cell HK2 were purchased from ATCC. HK2 cells were cultured in DMEM/F12 medium (Gibco, USA) containing 10% fetal bovine serum (Excell, China), renal cell carcinoma cells 786O, OSRC2, 769P, and ACHN were cultured in RPMI 1640 medium (Gibco, USA) containing 10% fetal bovine serum, and CAKI2 cells were cultured in McCoy’s 5A medium (Procell, China) containing 10% fetal bovine serum. All cell lines were incubated at 37 ° with 5% CO_2_.

Primary antibody against pan Keratin was bought from Cell Signaling Technology (Danvers, USA), Fluorescein (FITC)–conjugated Affinipure Goat Anti-Mouse IgG(H + L) was bought from Proteintech Group Inc. (USA).

### Animal experiments and clinical samples

Animal experiments were performed with the approval of Animal Care and Use Committee of the Second Affiliated Hospital of Xi’an Jiaotong University. Athymic BALB/c nude male mice (6 weeks old) were purchased from Beijing Vital River Laboratory Animal Technology Co., Ltd. (Beijing, China). 786O cells were resuspended in PBS to 5*10^6/ml, and 100ul of the cell suspension was injected into the tail vein of nude mice as described in previous literature. Six weeks after cell inoculation, the tumorc cells injected mice were sacrificed by cervical dislocation following anesthesia, blood was collected by cardiac puncture. Lymphocyte isolation solution (Solarbio, China) was used to remove red blood cells and plasma from the blood, and the remaining cells were resuspended in RPMI 1640 medium and seeded on cell culture plates. The solution was changed every 24 h until cell clones appeared, which was the CTC cell line.

We collected 15 pairs of ccRCC tumor tissue and normal control tissue samples at the department of urology, the second affiliated hospital of Xi’an Jiaotong University. All patients submitted informed consent for tissue use. Our studies involving human participants were in accordance with the ethical standards of the relevant guidelines and regulations. The study protocol was approved by the Ethics Committees of the second affiliated hospital of Xi'an Jiaotong University.

### Immunofluorescence

The adherent cells were washed with PBS, then fixed with 4% paraformaldehyde for 15 min and washed with PBS. Cells were treated with 0.5% Triton x-100 for 20 min at room temperature. After blocking with goat serum, the primary antibody was incubated overnight. Then the cells were incubated with secondary antibody for 1 h, and the nuclei were stained with DAPI. Finally, a fluorescence microscope was used to observe the results.

### Gene expression analysis by qPCR and weatern blot

Total RNA was extracted from cells using the Trizol method and subsequently reverse transcribed to cDNA using the reverse transcription kit (abm, Canada). The mRNA expression level was detected by the qPCR kit (DiNing, China). The primer sequences of all genes were shown in Table 2. All reagents are used in strict accordance with the actual instructions. Relative genes expression analysis was performed using the 2 –△△CT, with GAPDH used as an internal control.

**Table 2:** PCR primer sequences of genes.

Genes	Forward primers	Reverse primers
TYROBP	CCGGAAACAGCGTATCACT	TAATACGGCCTCTGTGTGTTG
MMP9	GAACCAATCTCACCGACAGG	GCCACCCGAGTGTAACCATA
GAPDH	GAGTCAACGGATTTGGTCGT	GACAAGCTTCCCGTTCTCAG

Protein samples of cells and tissues were separated by 10% SDS-PAGE and then transferred to membranes. After blocking with 5% skim milk, the corresponding primary antibody was added and incubated overnight. After 1 h incubation with secondary antibody, BCL luminescent solution was used for detection.

### Statistical analysis

Statistical analyses were performed using GraphPad (8.0.1) and R (4.1.1) software, and two tailed student’s *t* test was used to assess statistical differences between two groups. *p* < 0.05 was considered statistically significant.

### Ethics approval and consent to participate

The study protocol was approved by the Ethics Committees of the second affiliated hospital of Xi’an Jiaotong University. Animal experiments were performed with the approval of Animal Care and Use Committee of the Second Affiliated Hospital of Xi’an Jiaotong University. All animal experiments were performed in accordance with ARRIVE guidelines. All patients submitted informed consent for tissue use. All procedures performed in studies involving human participants were in accordance with the ethical standards of the 1964 Helsinki declaration, and its later amendments or comparable ethical standards.

## Results

### Identification of DEGs in CTCs

**Figure 1:**
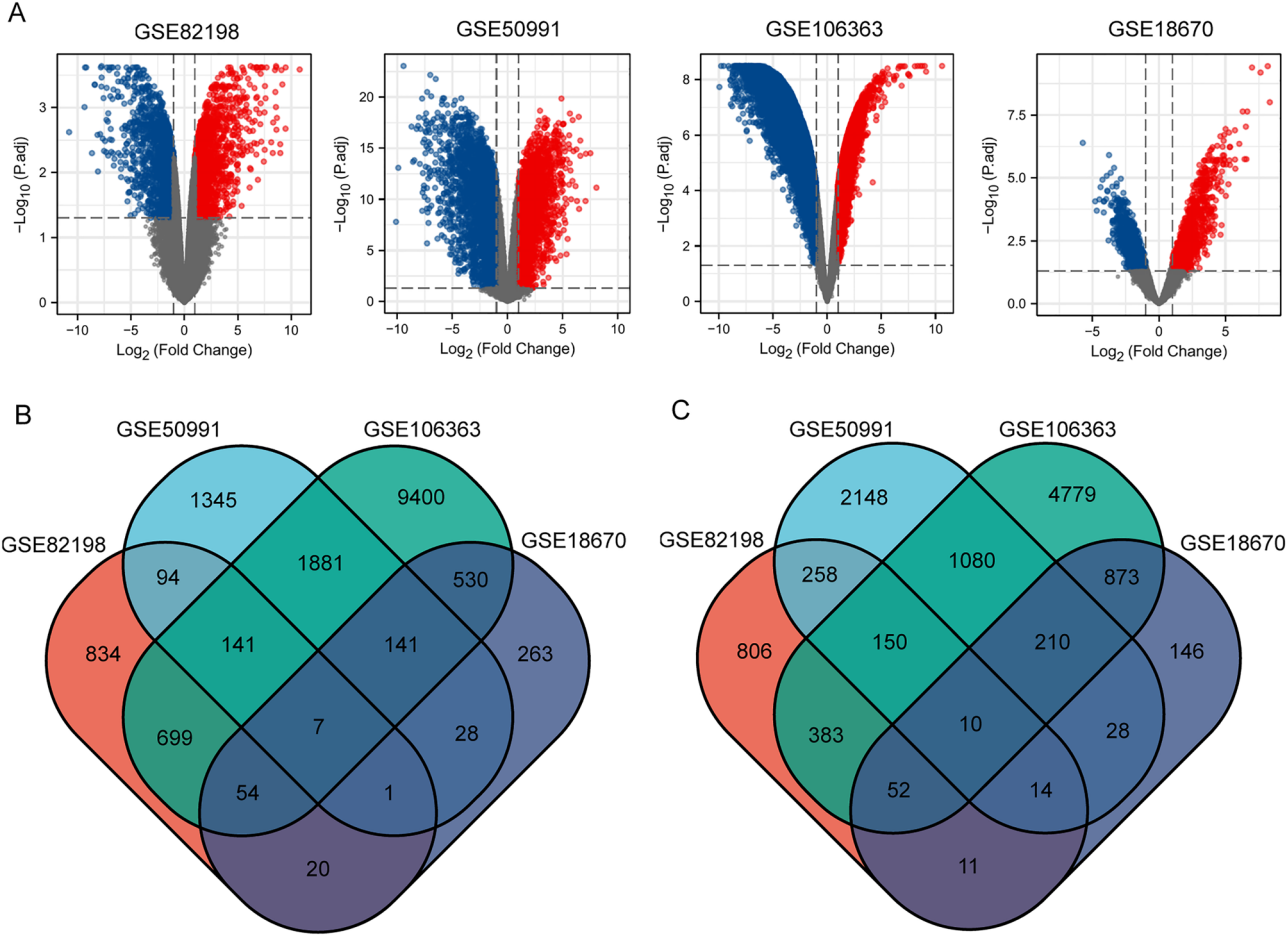
Differentially expressed genes in CTCs. (**A**) Differentially expressed genes in different datasets. (**B**) Venn diagram of up-regulated DEGs in CTCs. (**C**) Venn diagram of down-regulated DEGs in CTCs. The red and blue dots represent up-regulated and down-regulated genes, respectively. The threshold for fold change > 1.0 and a corrected *p*-value of < 0.05. Black points represent genes with no significant difference. *GEO* gene expression omnibus, *DEGs* differentially expressed genes.

**Figure 2:**
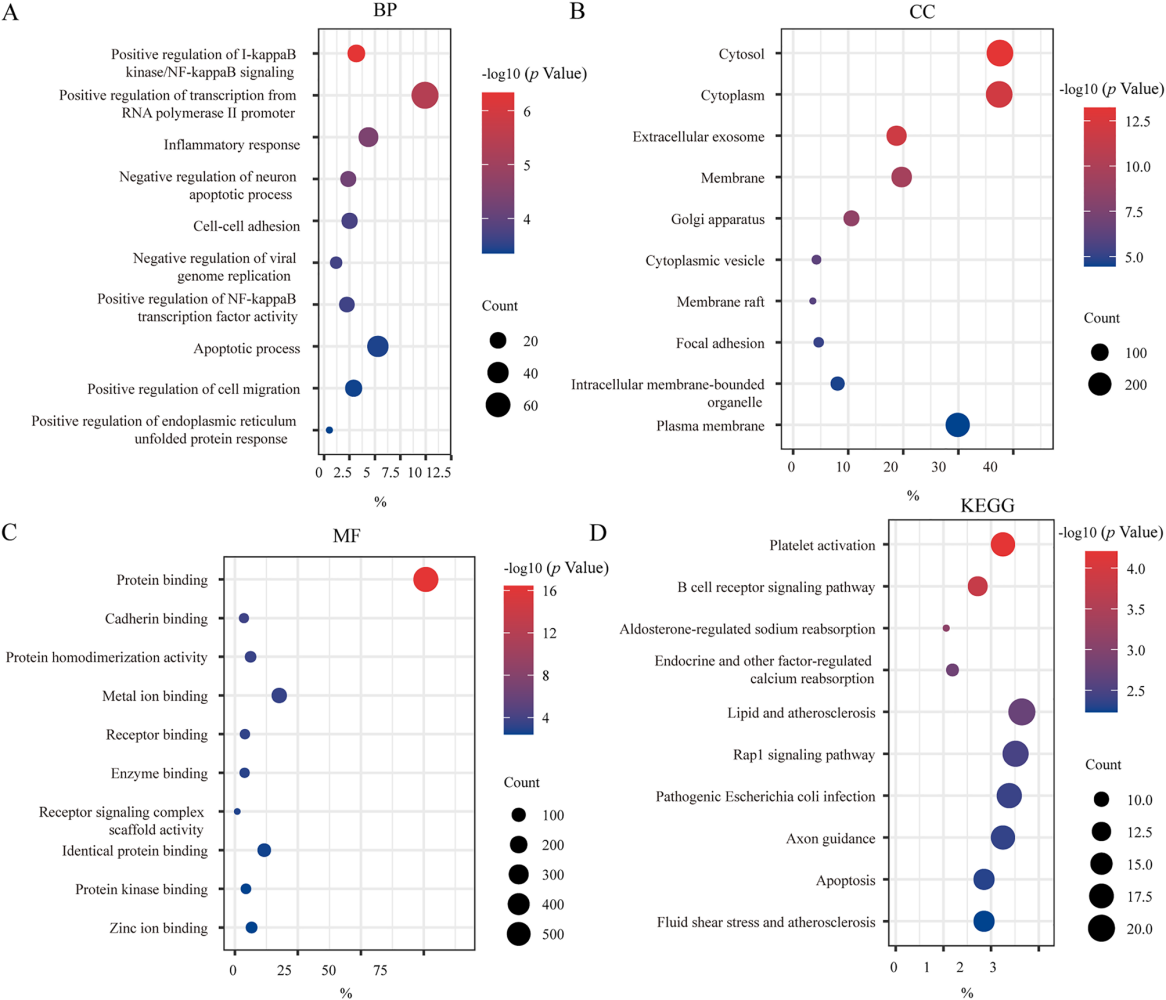
DEGs functional enrichment analysis. (**A**) BP biological process, (**B**) CC cellular component, (**C**) MF molecular function, (**D**) KEGG pathway.

We included 4 ccRCC GEO profile datasets in the study. The details of GSE18670, GSE50991, GSE82198, and GSE106363 are shown in the table (Table 1). The volcano map shows the DEGs in each dataset (Fig. 1A). We used the Venn diagram method to obtain the intersection of up-or down-regulated DEGs in the four data sets (Fig. 1B,C). We selected genes that were differentially expressed in more than three data sets. Finally, 344 up-regulated genes and 436 down-regulated genes were included. Next, we aimed to explore the biological processes associated with these DEGs. The DAVID database was used to annotate these robust DEGs, and GO and KEGG analyses were performed for these DEGs. In the BP part of GO analysis, DEGs are mainly enriched in the ‘Positive regulation of transcription from RNA polymerase II promoter’ and ‘Apoptotic process’ (Fig. 2A). The results of CC showed that DEGs were mainly concentrated in the ‘Cytosol’ and ‘Cytoplasm’(Fig. 2B). The main molecular function performed by DEGs was ‘Protein binding’(Fig. 2C). Through KEGG analysis, we learned that DEGs were mainly involved in ‘Platelet activation’, ‘B cell receptor signaling pathway’, ‘Lipid and atherosclerosis’ as well as ‘Biosynthesis of antibiotics’ (Fig. 2D). According to the results of these enrichment analyses, we found that the biological functions of these DEGs involved in apoptosis regulation and immune cell interaction were closely related to the onset and recurrence of the tumors.

### Hub genes selection and analysis

**Figure 3:**
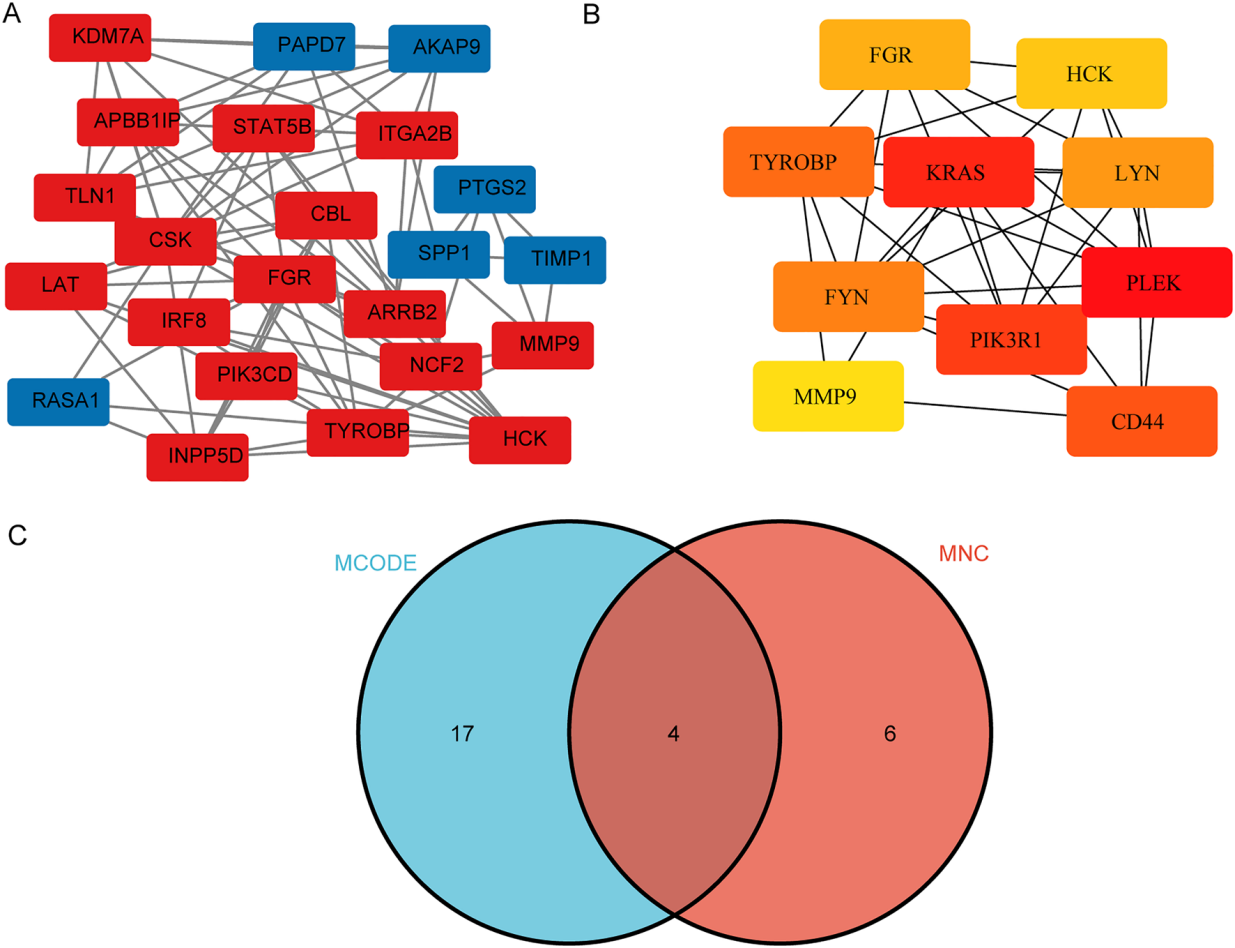
PPI network construction and analysis of key genes. (**A**) The subnetwork with the highest score. (**B**) The hub genes network. (**C**) The overlapped key genes.

**Figure 4:**
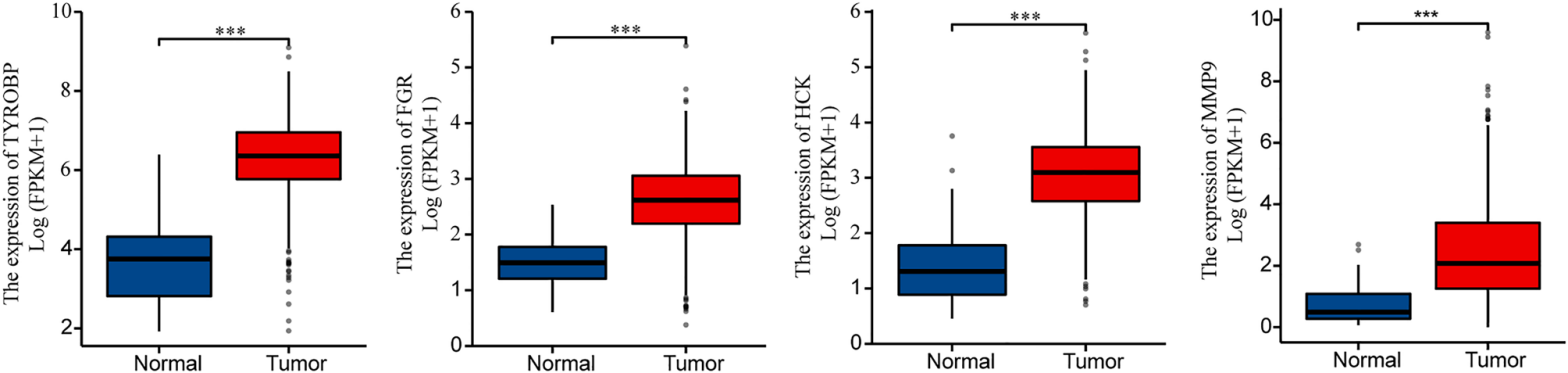
Expression of key genes in ccRCC. *p < 0.05; **p < 0.01; ***p < 0.001.

**Figure 5:**
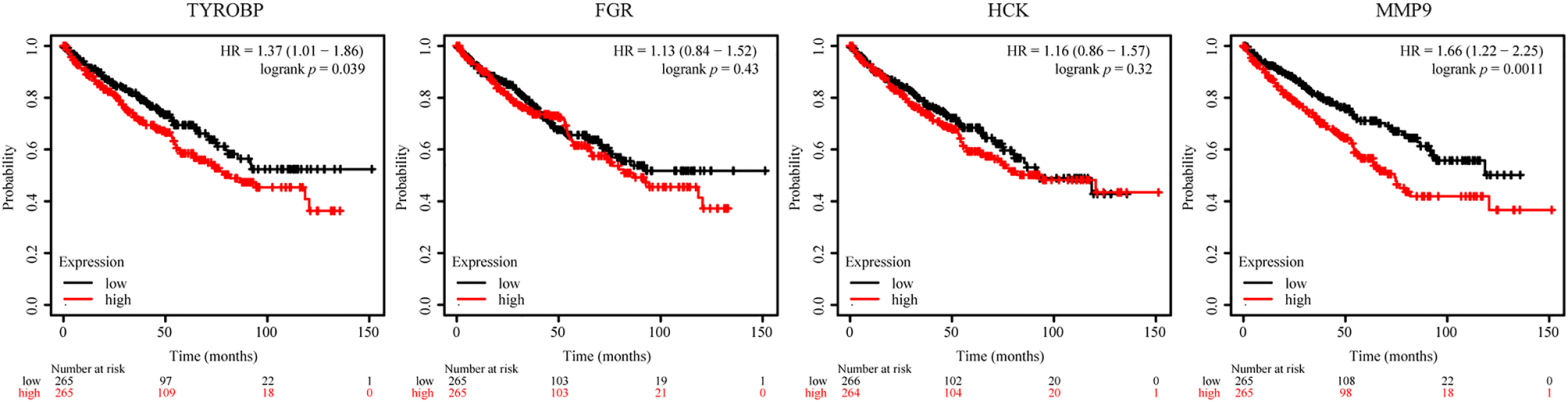
Relationship between key genes and ccRCC OS. *OS* overall survival, *HR* hazard ratio.

Using the Cytoscape software, a PPI network of DEGs was constructed. The MCODE plug-in was used to analyze the subnetwork, and selected the node genes in the subnetwork with the highest score (Fig. 3A). CytoHubba plug-in of Cytoscape software was used to identify hub genes of ccRCC according to the MNC algorithms (Fig. 3B). The overlapped hub genes in the results of the two methods included 4 genes (TYROBP, FGR, HCK, and MMP9) (Fig. 3C). The expression levels and prognostic value of these 4 hub genes were studied in ccRCC. The expression levels of these 4 genes in ccRCC tissues were higher than those in normal tissues (Fig. 4). Furthermore, high expression of TYROBP, and MMP9 suggested shorter OS in ccRCC patients, while the expression levels of the remaining 2 genes were not associated with OS in ccRCC patients (Fig. 5).

### Gene enrichment analysis

**Figure 6:**
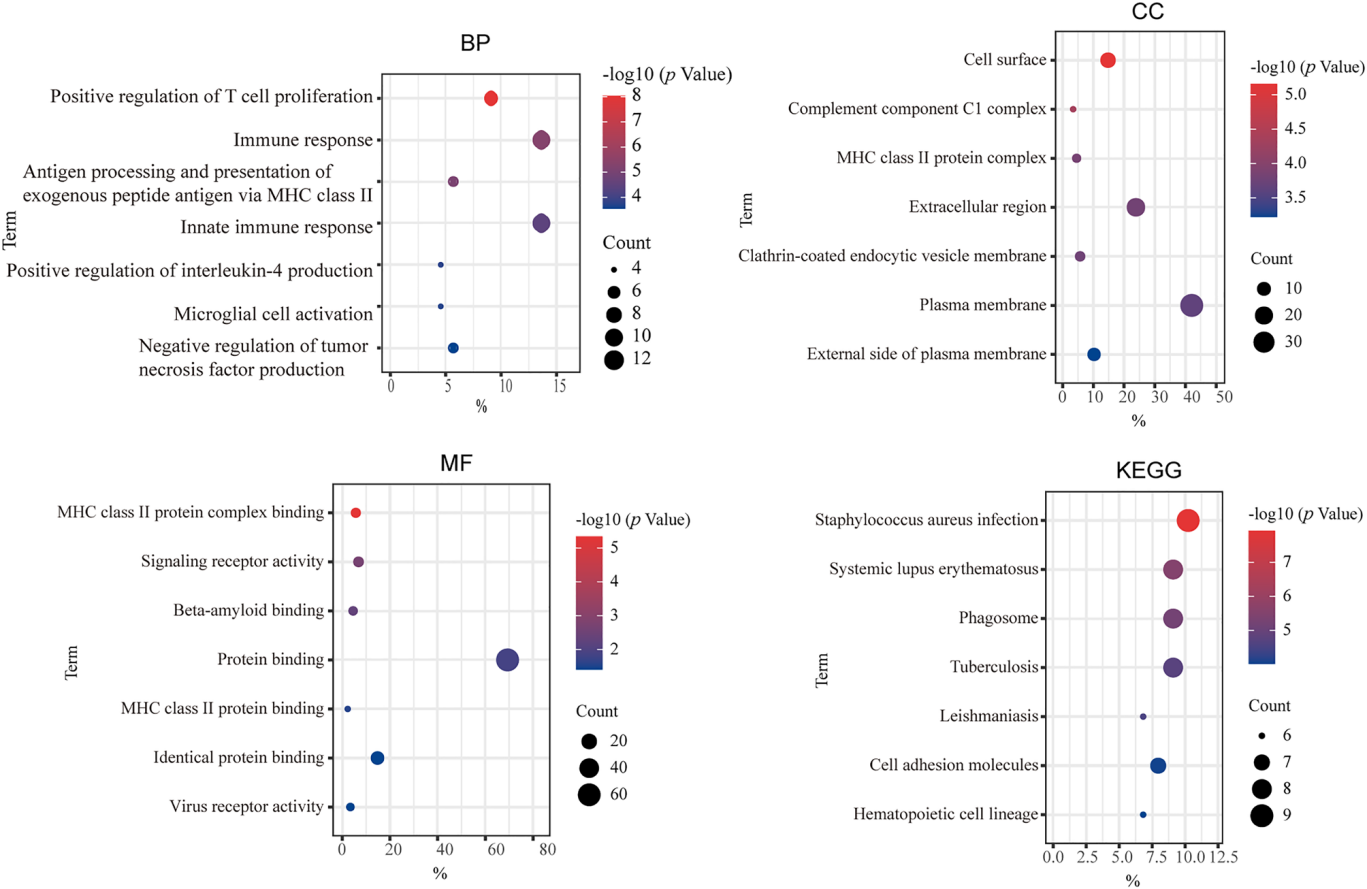
Enrichment analysis of genes associated with key genes.

To deeply understand the molecular mechanism of these 2 genes involved in the regulation of ccRCC disease progression, we used the GEPIA database to obtain the top 50 genes with the highest correlation with the expression of these two genes in ccRCC for functional analysis. The results showed that these genes were mainly involved in ‘Immune response’, ‘Innate immune response’, ‘Positive regulation of T cell proliferation’, ‘staphylococcus aureus infection’, and ‘systemic lupus erythematosus’ (Fig. 6). These biological processes are mostly related to tumor immune infiltration, suggesting that the genetic changes of CTCs in ccRCC affect tumor progression by regulating the tumor immune microenvironment.

### Relationship between prognostic DEGs and immune characteristics

**Figure 7:**
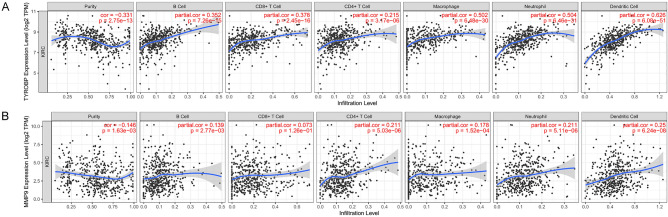
Correlation analysis of key genes expression with tumor immune-associated cells. (**A**,**B**) Relationship between MMP9 and TYROBP expression and the level of tumor-associated immune cell infiltration in ccRCC.

In recent years, immune checkpoint inhibitors are considered to be effective against a wide range of tumors, the research on tumor immune checkpoints has provided many important targets for tumor immunotherapy. According to the TIMER database, we found that these two genes are strongly associated with the infiltration levels of various immune cells in ccRCC. In particular, the expression of TYROBP and MMP9 showed a significant positive correlation with the infiltration level of various immune cells. (Fig. 7A,B).

**Figure 8:**
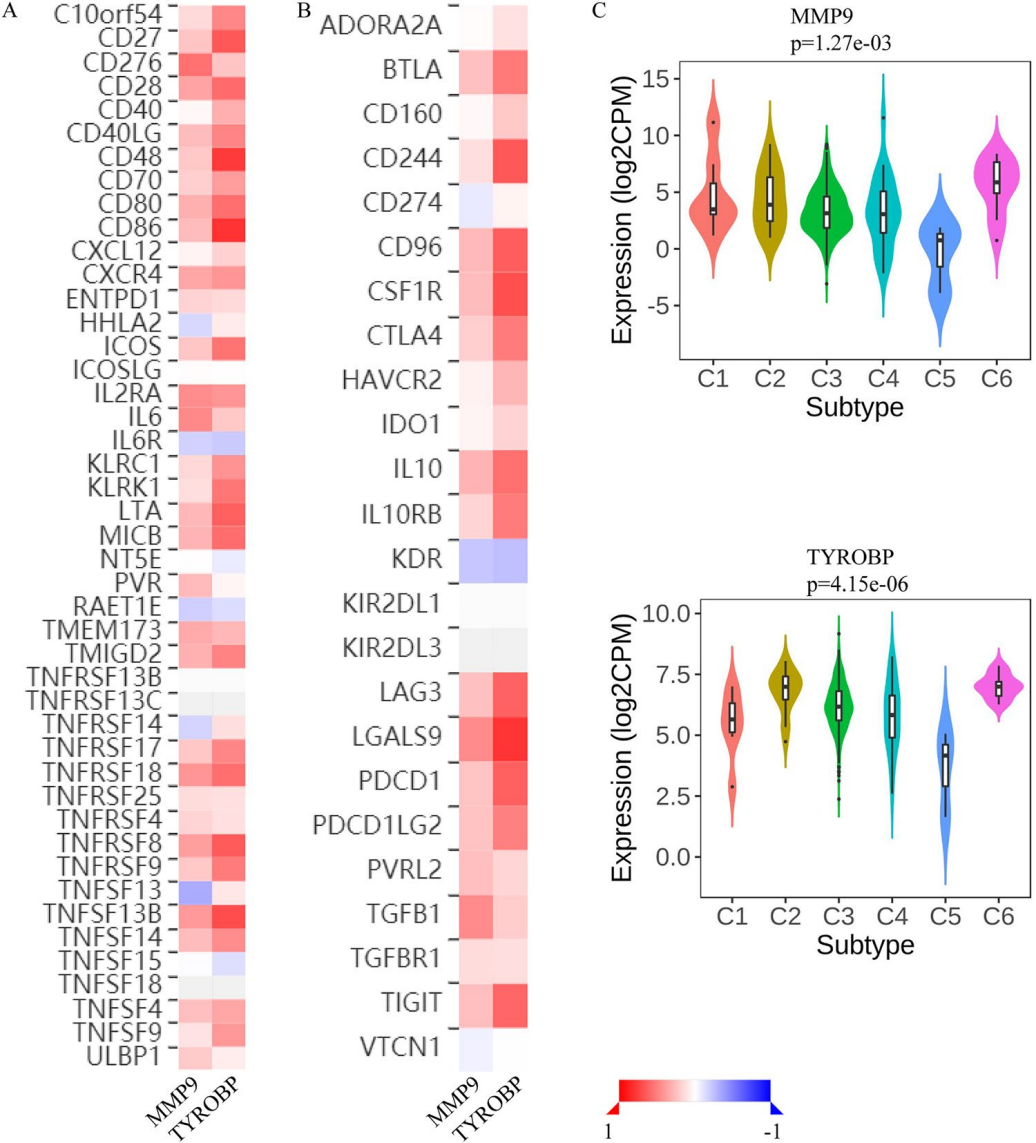
Immunocorrelation analysis of key genes. (**A**) Correlations between key genes and immune stimulators. (**B**) Correlations between key genes and immune inhibitors. (**C**) Association between key genes and immune subtypes of ccRCC. Red represents a positive relationship and blue represents a negative relationship.

The results of the TISIDB database showed that these 2 genes were closely related to immune-related markers of ccRCC (Fig. 8A,B). The immune stimulators that showed the strongest positive correlation with MMP9 and TYROBP genes were CD276 and CD86 respectively (SFig. 1A). TNFSF13 and IL6R had the strongest negative correlation with these 2 genes (SFig. 1B). Among the immune inhibitors, TGFB1 and LGALS9 showed the strongest positive correlation with these 2 genes (SFig. 1C), while KDR showed the strongest negative correlation (SFig. 1D). In addition, the analysis results of immune subtypes showed that the expression of these 2 genes was significantly different among different immune subtypes of ccRCC (Fig. 8C), which indicated that these genes were widely involved in the regulation of immune microenvironment of ccRCC.

### CTCs isolation and validation

**Figure 9:**
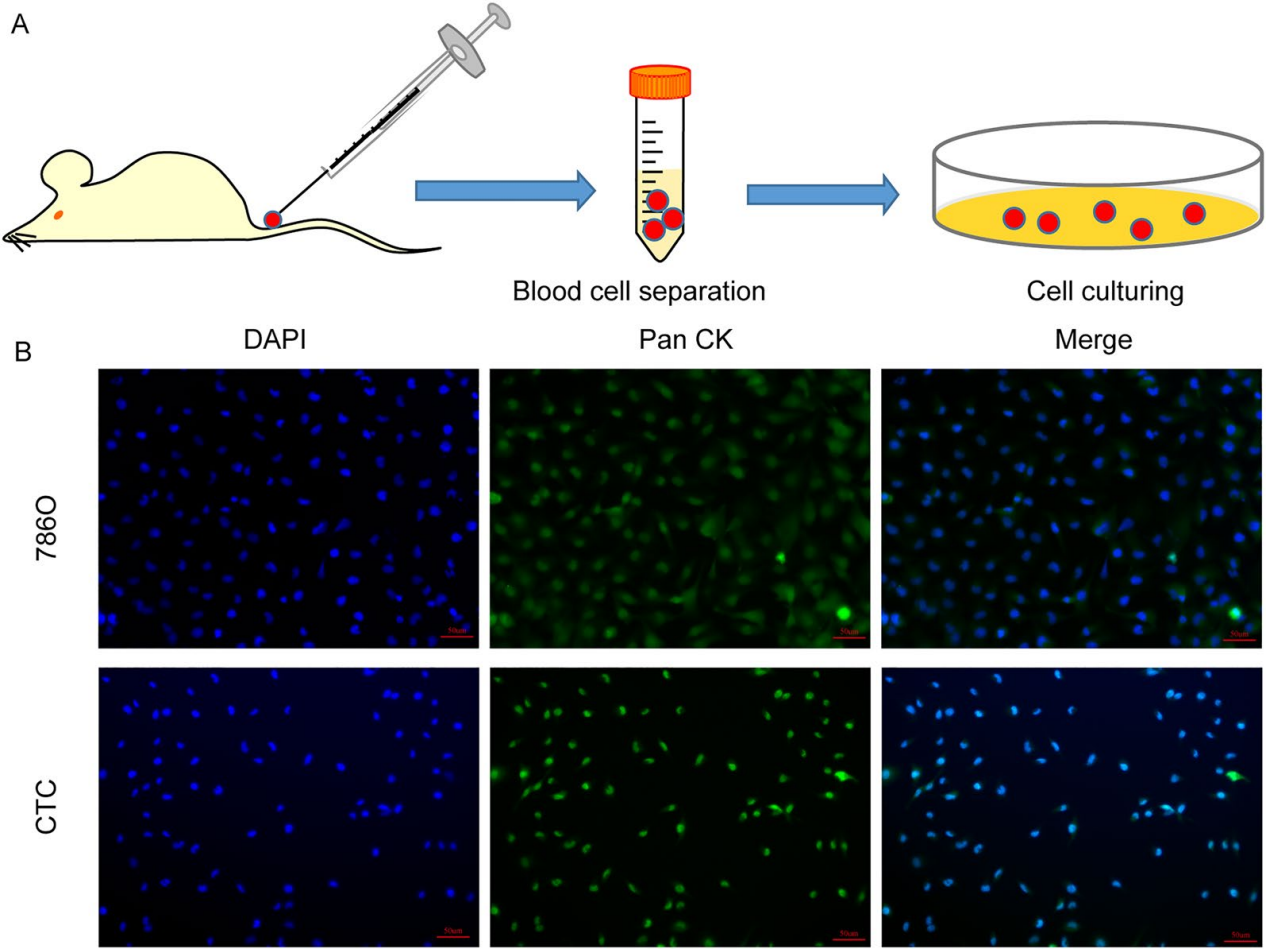
CTCs isolation and validation. (**A**) Schematic of the in vivo selection method. (**B**) The isolated cells were identified as CTCs by immunofluorescent staining.

In this study, we used in vivo selection method to construct CTC cells according to previous reports^16,17^ (Fig. 9A). To verify that our method resulted in ccRCC CTC cells, we stained the obtained cells with antibodies to CK, a protein widely expressed in CTC cells. The results of immunofluorescence showed that the cells we isolated were positive for CK, indicating that these cells were CTCs (Fig. 9B).

### The expression of key genes

**Figure 10:**
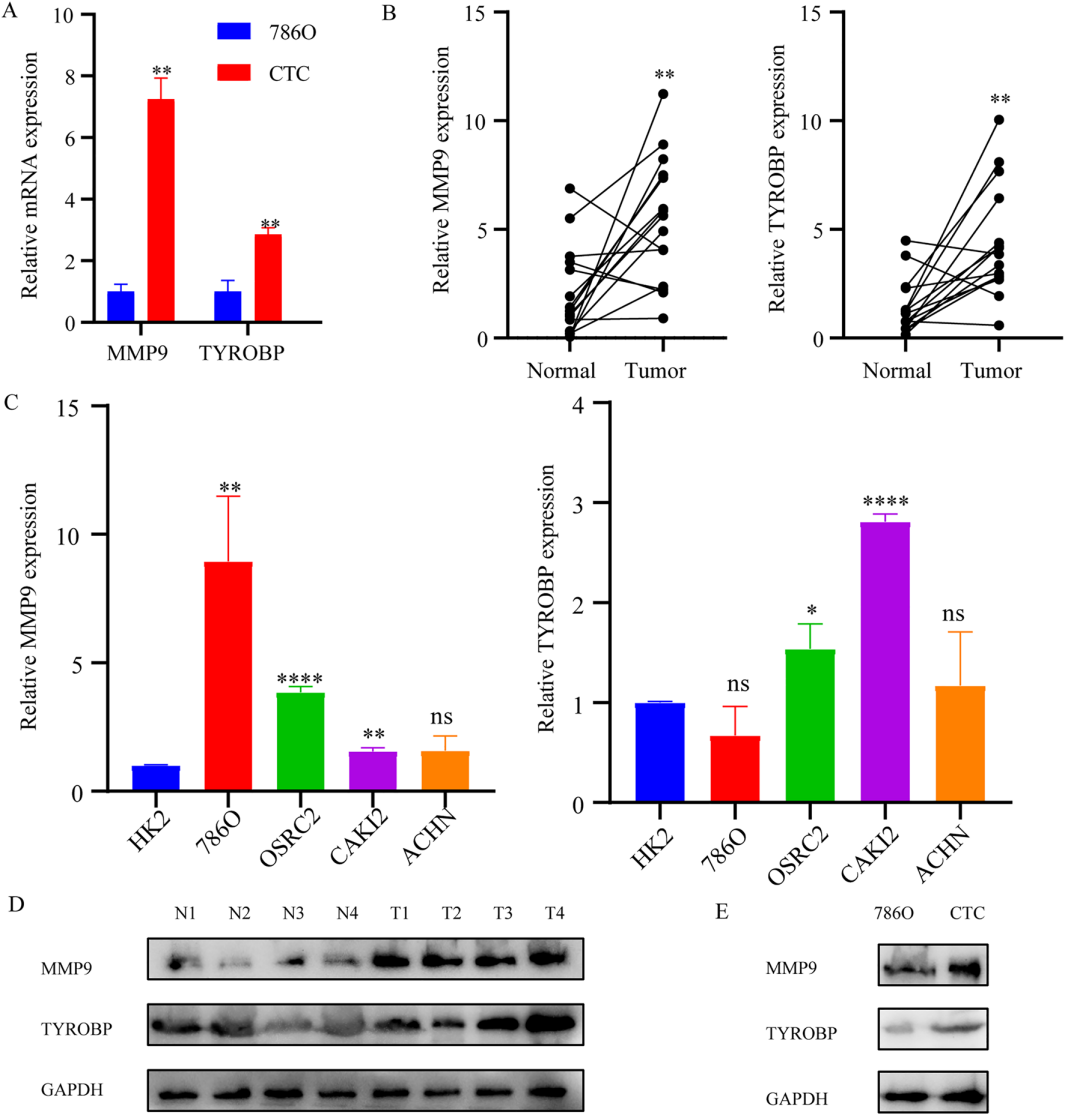
The expression of key genes. (**A**) The expression levels of MMP9 and TYROBP in CTCs. (**B**) The mRNA expression levels of MMP9 and TYROBP in renal cancer tissues. (**C**) The mRNA expression levels of MMP9 and TYROBP in renal cancer cell lines. (**D**) Western blot analyses of MMP9 and TYROBP expression in 4 representative tumors and paired adjacent tissues. (**E**) Western blot analyses of MMP9 and TYROBP expression in CTCs. *p < 0.05; **p < 0.01; ***p < 0.001.

Subsequently, we used the constructed CTCs cell model of ccRCC to detect the expression of these two genes. The results showed that the expression levels of MMP9 and TYROBP in CTCs were significantly increased compared with the 786O cell line (Fig. 10A). We also examined the mRNA expression of these 2 genes in renal cancer tissues, and the results showed that the mRNA expression levels of MMP9 and TYROBP in ccRCC tissues were significantly increased (Fig. 10B). The expression levels of these 2 genes in renal cancer cell lines 786O, OSRC2, and CAKI2 were significantly higher than that in normal control HK2 cell lines (Fig. 10C). Previous results of immunocorrelation analysis showed that MMP9 and TYROBP were most closely correlated with the level of immunoinfiltration in ccRCC, and western blot experiment was used to detect the expression of these two molecules. The results showed that these two molecules were significantly upregulated in ccRCC tissues compared with adjacent normal tissues (Fig. 10D). The protein expression levels of these 2 genes in CTCs were significantly increased compared with the 786O cell line (Fig. 10E).

## Discussion

Cancer is a complex disease involving multiple genes. It is widely believed that a tumor is not composed of a single type of cell population, but consists of subsets of cells with different genes and phenotypes^18^. Tumor heterogeneity can result from genetic or non-genetic changes, such as changes in the expression of tumor molecules or different external stimuli^19^. After the tumor cells escape from the extracellular matrix and enter the blood circulation, most of the tumor cells die due to the shear force of the intravascular fluid, the anoikis, and the killing effect of immune cells^20,21^. Only a few of the tumor cells survive. These surviving CTCs are a group of genetically and phenotypically highly heterogeneous cells, often with specific biological characteristics to adapt to the new environment^22^. In this study, we analyzed the key genes with altered expression in ccRCC CTCs, explored the association between these genes and the progression of ccRCC, and finally determined that MMP9 and TYROBP played a key role in the development of ccRCC CTCs.

After entering the peripheral blood circulation, single tumor cells will be attacked by the body’s immune cells and undergo apoptosis. Immune cells are the main reason that restricts the survival of CTCs in blood circulation^23^. A large number of CTCs die due to the killing effect of immune cells, and only a small part of CTCs have the ability to escape the killing of immune cells and survive in the blood circulation^24^. These surviving CTCs evade immune killing by regulating their gene expression, downregulating the ligands that react with NK cells, and taking refuge from other cellular components^25^.

In our study, we also found that DEGs in CTCs were widely involved in various immune processes in the body. The results of gene enrichment analysis of DEGs showed that these DEGs affected 'Platelet activation' and 'B cell receptor signaling pathway’. Genes associated with MMP9 and TYROBP expression in ccRCC were involved in the regulation of ‘Immune response’ and ‘Innate Immune response’.

Immunotherapy is an effective treatment for advanced tumors^26^. Recently, tumor-associated immune cells have been widely concerned because of their important role in tumor progression and treatment^27^. The specific mechanism of tumor-associated immune cells in tumors is often extremely complex. Further exploration of immune infiltration of tumors is helpful to deepen our understanding of the progression of tumor diseases.

Our study found that among the two key genes of CTCs in ccRCC, MMP9 and TYROBP showed a significant positive correlation with the infiltration levels of B cells, CD4 + T cells, CD8 + T cells, macrophages, and other immune cells in ccRCC. The expression of these genes is significantly correlated with various immune markers of ccRCC, and the expression of these genes is different among different immune subtypes of ccRCC. These findings suggest that these 2 genes may affect the survival of CTCs by regulating the tumor immune microenvironment of ccRCC.

Previous studies have shown that the 2 genes MMP9 and TYROBP regulate the progression of a variety of tumor diseases. MMP9 is a type of matrix metalloproteinase that breaks down the extracellular matrix. The abnormal expression of MMP9 is related to the occurrence of many diseases such as cancers. Inhibitors of MMP9 can be used to treat cancers^28^. Hong et al. found that the positive expression level of MMP9 in CTCs of colorectal cancer was an independent prognostic factor for progression-free survival^29^. In addition, MMP9 also deeply regulates the immune microenvironment of tumors. The study has shown that MMP9 promotes tumor progression by regulating tumor immune infiltration in ccRCC^30^. TYROBP (Transmembrane Immune Signaling Adaptor TYROBP) contains an immunoreceptor tyrosine-based activation motif^31^. Lu et al. found that the high expression of TYROBP could affect the immune infiltration of glioma and predict poor prognosis^32^.

In this study, we found that DEGs between CTCs and primary tumors were mainly involved in the regulation of tumor immunity and the survival process of CTCs in blood circulation. At the same time, we verified the possibility of MMP9 and TYROBP as therapeutic and prognostic targets of ccRCC by constructing a CTCs model and detecting the gene expression of ccRCC tissue samples collected. However, we included a small number of ccRCC clinical specimens and lacked genetic testing for clinical CTCs. In the following study, we will continue to collect and detect the expression of CTCs genes in the peripheral blood of ccRCC patients and verify the function of these key genes through molecular and cellular experiments.

## Conclusion

We found DEGs between CTCs and primary tumor cells based on data from the GEO database. Through the correlation analysis and enrichment analysis of these genes, the key genes in ccRCC were screened. GO and KEGG enrichment analysis suggested that these genes mainly affected the survival of CTCs by regulating the tumor immune microenvironment. Finally, we used the in vivo screening method to construct the ccRCC CTCs model and verified the expression of key genes at the cellular and tissue levels, proving that these genes can be used as potential therapeutic and prognostic targets of ccRCC.

## Supplementary Information

Supplementary Legends.

Supplementary Figure 1.

Supplementary Figures.

## Data Availability

The datasets analyzed during the current study are available in the TCGA database (https://tcga-data.nci.nih.gov/tcga/) and GEO database (https://www.ncbi.nlm.nih.gov/geo/). All the data were available from the corresponding authors for reasonable request.
