# Supplementary material for: MMP9 and TYROBP affect the survival of circulating tumor cells in clear cell renal cell carcinoma by adapting to tumor immune microenvironment
Source: Sci Rep. 2023 Apr 28;13:6982. doi: 10.1038/s41598-023-34317-2 (PMC10147606; doi:10.1038/s41598-023-34317-2)
Supplement: Supplementary file 1 — Supplementary Legends. [file 41598_2023_34317_MOESM1_ESM.docx]

Supplement figure legend

SFigure 1. Correlations between key genes and immune markers. (A) The immune stimulators showed the strongest positive correlation with MMP9 and TYROBP. (B) The immune stimulators showed the strongest negative correlation with MMP9 and TYROBP. (C) The immune inhibitors showed the strongest positive correlation with MMP9 and TYROBP. (D) The immune inhibitors showed the strongest negative correlation with MMP9 and TYROBP.
