# Supplementary figures and images for: MMP9 and TYROBP affect the survival of circulating tumor cells in clear cell renal cell carcinoma by adapting to tumor immune microenvironment
Source: Sci Rep. 2023 Apr 28;13:6982. doi: 10.1038/s41598-023-34317-2 (PMC10147606; doi:10.1038/s41598-023-34317-2)

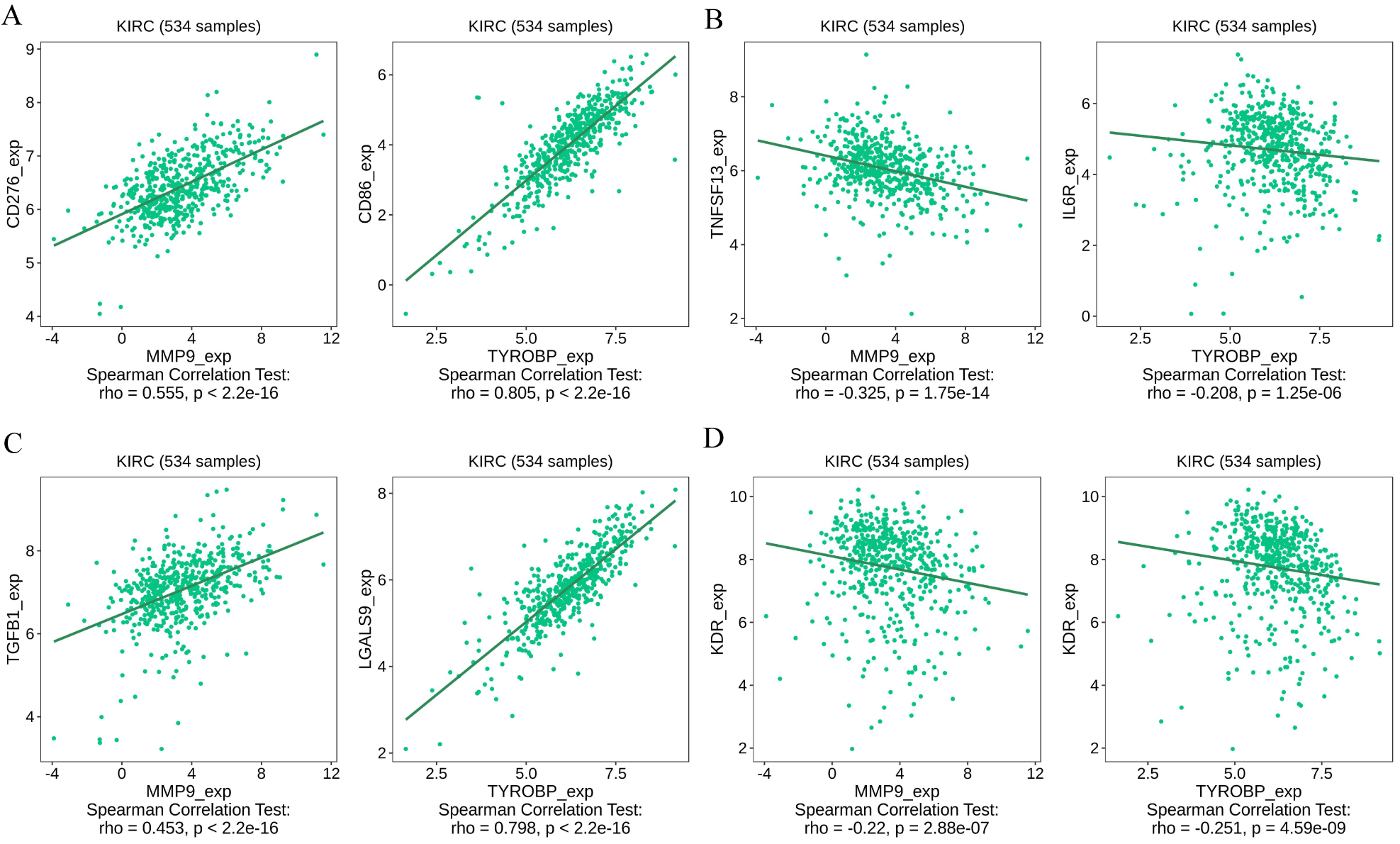

Supplement: Supplementary file 2 — Supplementary Figure 1. [file 41598_2023_34317_MOESM2_ESM.tif]
