# Supplementary material for: MMP9 and TYROBP affect the survival of circulating tumor cells in clear cell renal cell carcinoma by adapting to tumor immune microenvironment
Source: Sci Rep. 2023 Apr 28;13:6982. doi: 10.1038/s41598-023-34317-2 (PMC10147606; doi:10.1038/s41598-023-34317-2)
Supplement: Supplementary file 3 — Supplementary Figures. [file 41598_2023_34317_MOESM3_ESM.pdf]

A.MMP9

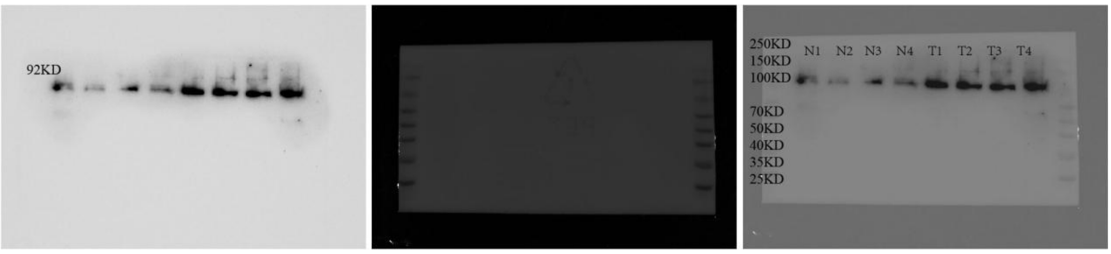

B.TYROBP

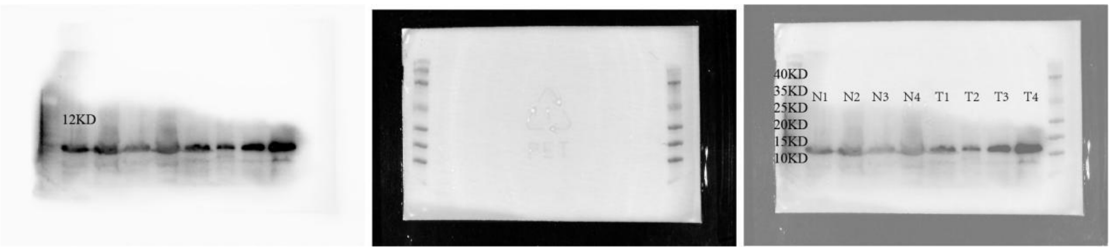

C.GAPDH

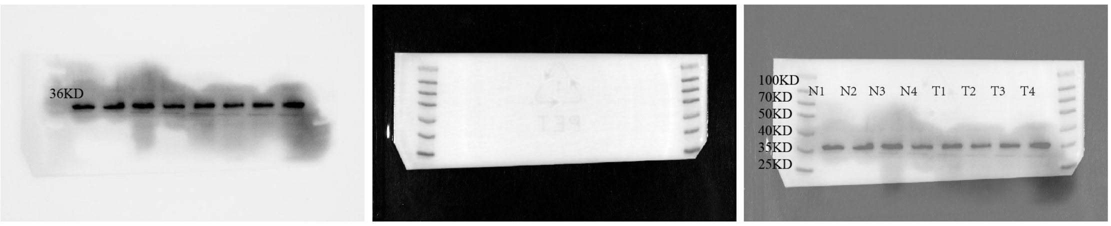

Supplement figure 2. Raw immunoblot data for images in Fig. 10D. (A).Immunoblot anti-MMP9 of ccRCC tissue samples. (B).Immunoblot anti-TYROBP of ccRCC tissue samples. (C).Immunoblot anti-GAPDH of ccRCC tissue samples.

A.MMP9

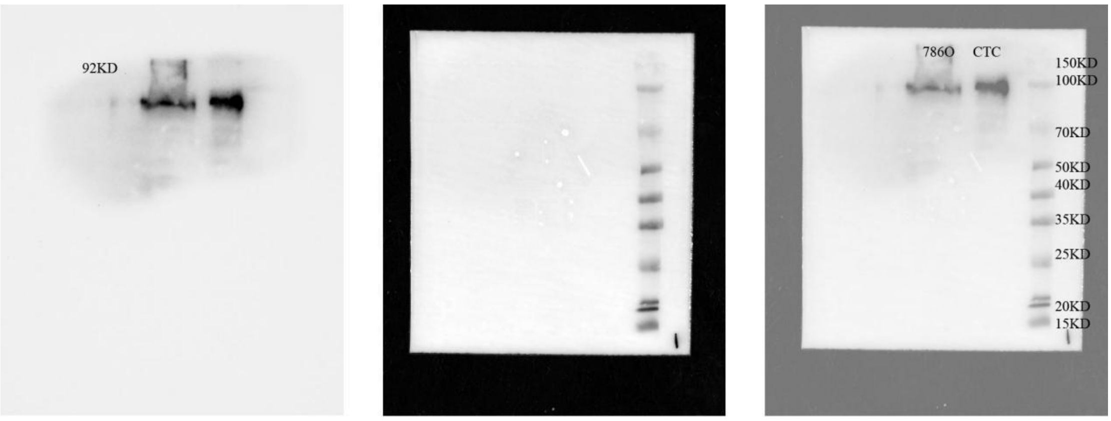

B.TYROBP

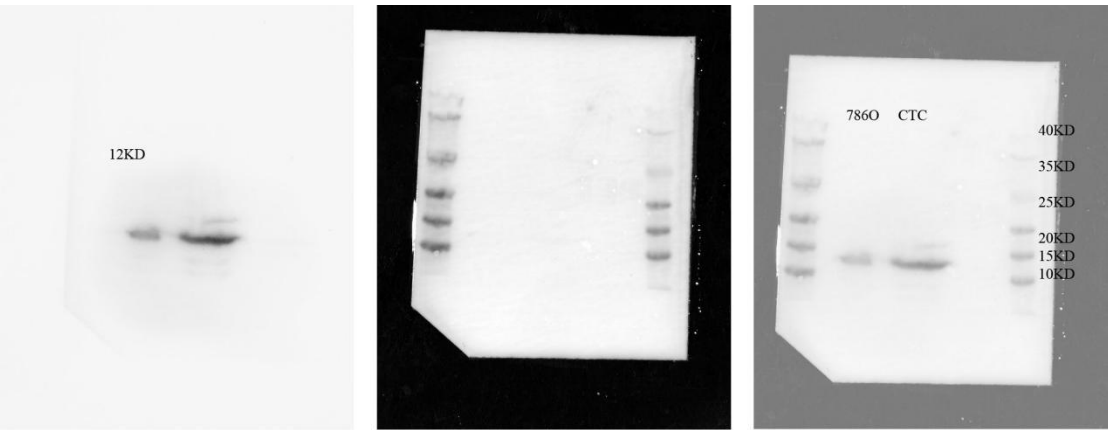

C.GAPDH

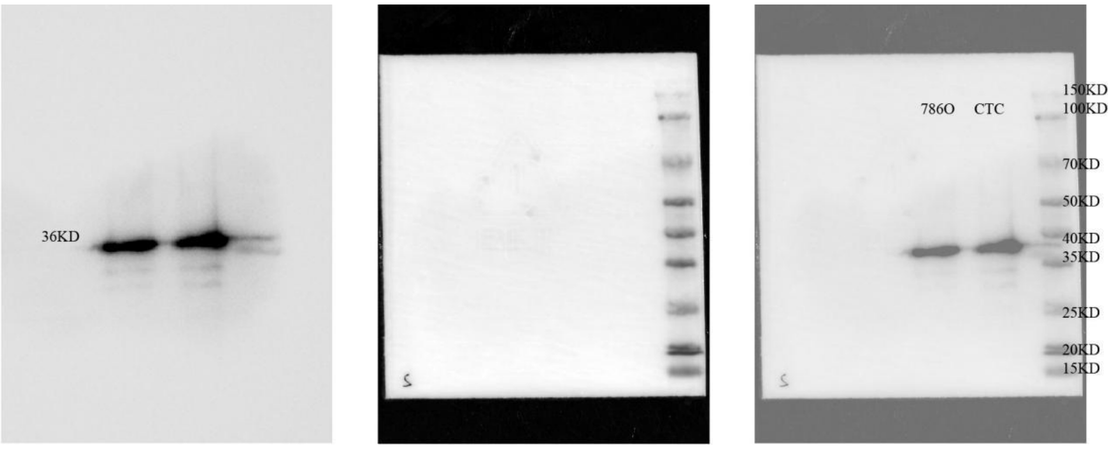

Supplement figure 3. Raw immunoblot data for images in Fig. 10E. (A).Immunoblot anti-MMP9 of CTC. (B).Immunoblot anti-TYROBP of CTC. (C).Immunoblot anti-GAPDH of CTC.
